# In-Group Conformity Sustains Different Foraging Traditions in Capuchin Monkeys (*Cebus apella*)

**DOI:** 10.1371/journal.pone.0007858

**Published:** 2009-11-18

**Authors:** Marietta Dindo, Andrew Whiten, Frans B. M. de Waal

**Affiliations:** 1 Centre for Social Learning and Cognitive Evolution, School of Psychology, University of St Andrews, Fife, United Kingdom; 2 Living Links, Yerkes National Primate Research Center, Emory University, Atlanta, Georgia, United States of America; Università di Parma, Italy

## Abstract

**Background:**

Decades of research have revealed rich cultural repertoires encompassing multiple traditions in wild great apes, a picture crucially complemented by experimental simulations with captive apes. Studies with wild capuchin monkeys, the most encephalized simian species, have indicated a New World convergence on these cultural phenomena, involving multiple traditions and tool use. However, experimental studies to date are in conflict with such findings in concluding that capuchins, like other monkeys, show minimal capacities for social learning.

**Methodology/Principal Findings:**

Here we report a new experimental approach in which the alpha male of each of two groups of capuchins was trained to open an artificial foraging device in a quite different way, using either a slide or lift action, then reunited with his group. In each group a majority of monkeys, 8 of 11 and 13 of 14, subsequently mastered the task. Seventeen of the successful 21 monkeys discovered the alternative action to that seeded in the group, performing it a median of 4 times. Nevertheless, all 21 primarily adopted the technique seeded by their group's alpha male. Median proportions of slide versus lift were 0.96 for the group seeded with slide versus 0. 01 for the group seeded with lift.

**Conclusions/Significance:**

These results suggest a striking effect of social conformity in learned behavioral techniques, consistent with field reports of capuchin traditions and convergent on the only other species in which such cultural phenomena have been reported, chimpanzees and humans.

## Introduction

The study of culture in animals has its origin in field reports from primatology decades ago, describing ‘proto-cultural’ behavior in Japanese macaques on Koshima Island [1], [2]. In a provisioned troop, a juvenile female, Imo, began taking sweet potatoes presented on the sandy beach, and submerged the potatoes under water before eating. The gradual spread of this behavior, which became known as potato-washing, was documented for decades to reveal a very slow spread that began among related females, and eventually extended to many other family groups within the troop [3], [4]. The ‘cultural’ status of this celebrated case was later questioned because the spread appeared too slow to be explained by observationally based social learning [5], [6]. However, such critiques did not take into consideration the particularly despotic nature of macaque social structure [7], [8], [9]. Opportunities for social learning in this species were likely to have been limited by the level of social tolerance exhibited between ‘potato-washers’ and naïve observers [10].

Despite the rich behavioral data available from Koshima and other primate field sites since, we still know little about the ways in which traditions and other culturally acquired behaviors spread in wild populations of monkeys [11], [12]. For apes, there is evidence for group-specific foraging behaviors, as well as a substantial repertoire of tool-use behaviors and social conventions, which have been suggested to shed light on human cultural origins [13], [14]. These putative examples of wild ape culture have a much clearer connection than in monkeys to decades of experimental work demonstrating the observational learning skills of apes in captivity, in particular chimpanzees [15], [16], [17], [18], [19], [20]. By contrast, in an influential paper, Visalberghi and Fragaszy [21] argued that monkeys ‘do not ape’: in other words, the observational learning skills of monkeys do not lead to copying of others' behaviors. Fragaszy and Visalberghi (p. 24) [22] went on to more specifically state that monkeys “do not learn *from* each other”, rather they “learn *with* each other”. While the numerous examples these authors presented support the claim that monkeys appear to be weak social learners, this created a puzzling gap in explaining newly emerging evidence from the field that capuchin monkeys maintain social conventions and other group specific foraging traditions that are highly suggestive of social transmission [23], [24], [25], [26], [27]. How was it possible that such traditions would be maintained and spread in the absence of imitation or other forms of social transmission?

With new advances in experimental approaches we are beginning to see more convincing evidence that monkeys may copy other group members in more sophisticated ways than previously suggested. In the ‘two-action’ paradigm [28], two different methods of solution are possible, but only one is demonstrated to each subject. Several studies employing this approach have now provided evidence of monkeys copying what they see another do [29], [30], [31], [32], [33], [34], [35].

However, such evidence of social learning may be limited by social and physical contexts as well as by individual motivation [10], [36], [37]. Variation in motivation can be the most difficult to discern as it relates to multiple aspects of an individual's relationships within a group such as age, rank, relatedness and overall affiliation with others. This phenomenon has been described as *Bonding- and Identification-Based Observational Learning* (BIOL) by de Waal [38], referring to an intrinsically rewarding motivation to act like others with whom a close relationship exists. Indeed, Bonnie & de Waal [39] found that rewards are not essential for social learning in brown capuchin monkeys. Under this model, as with the social tolerance model suggested by Coussi-Korbel and Fragaszy [10], it is predicted that in this primate, which is marked by high levels of social tolerance (i.e. maintaining close physical proximity without aggression), learning opportunities are highest among those with the strongest social affiliations.

Dindo and colleagues [30] took due account of such social and motivational factors in a ‘transmission chain’ study in which alternative techniques for opening an artificial ‘Doorian Fruit’ (i.e. either slide a door open to obtain food or lift the same door) were seeded in one individual in each of two groups, and the transmission of these alternatives then tracked along chains in which the observer of the first model later became a model for a third, and so on along the chain. This study controlled for social ties between the model and observer at each step along the chain, checking for social tolerance in joint feeding opportunities prior to the test condition. Results showed high levels of copying fidelity, contrasting with the previous findings for capuchins reviewed above.

However, although this study identified a condition in which monkeys accurately copied the foraging activities of a conspecific, it did not show whether the behavior would spread in the natural, full group context, where all members have access to the foraging apparatus. Such an ‘open group’ scenario should provide a more ecologically valid picture of the spread of socially-transmitted behaviors as it may occur in the wild. If capuchin monkeys are able to learn a method for foraging by observing other group members, then we predict that alternative techniques will spread with significant fidelity within different groups. Here we report the first such open diffusion experiment to be completed in monkeys. The alpha male of each of two new groups of capuchins was trained to open the Doorian using either the slide or lift technique, and subsequently was reunited with his group. We then investigated the potential spread of this new behavior in each group.

## Results

**Figure 1:**
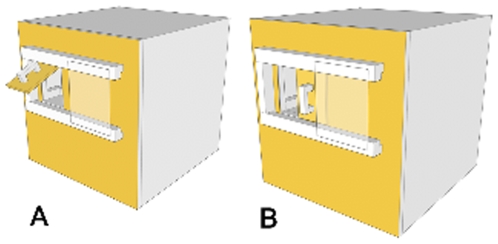
Foraging apparatus. The ‘Doorian Fruit’ presented two distinct methods for extracting food from the apparatus. The same door could either be lifted (a) or slid (b) open in order to reveal a food tray.

**Figure 2:**
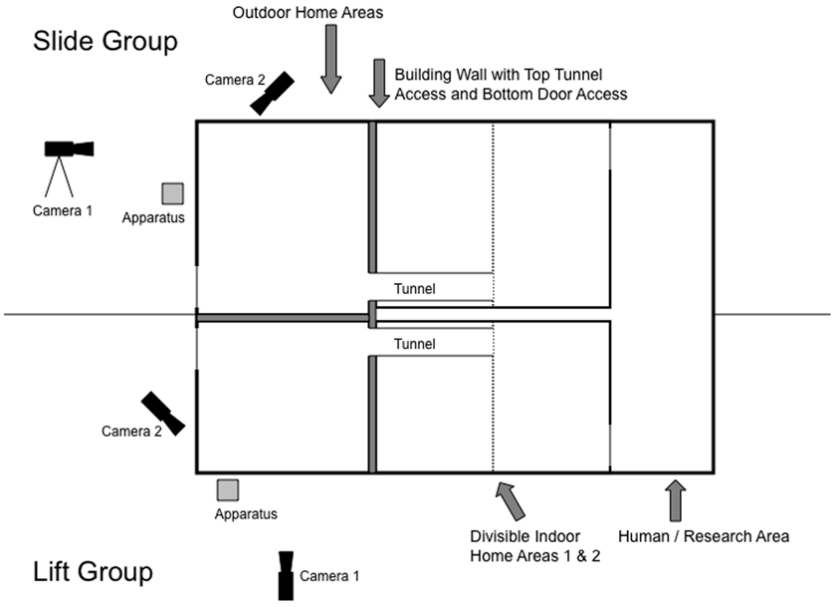
Testing Areas. A floor plan showing the outdoor areas for each group and the locations of video cameras and Doorian foraging device. Thick partitions are opaque, so each group cannot see the other.

Following the training of the model with the Doorian (Figure 1), the Doorian was presented to each group in their separate outside enclosures (Figure 2). In an initial Observation Phase of five, daily sessions, the alpha male model was able to monopolize the Doorian for the 9–10 minutes it took to complete 50 trials. Thus, other group members could only observe the model in this phase. We found that each model demonstrated only his respective trained method, lift or slide, although both methods were always possible.

In the group seeded with the lift technique (henceforth the ‘Lift’ group: N = 15 monkeys, see Table 1), only the highest ranking group members (SN, ST, SL, SM) and the two youngest group members (GN, BK) were able to observe the model within one meter of the apparatus. In the group seeded with slide (henceforth the ‘Slide’ group: N = 12 monkeys, see Table 1), a low ranking female (LL) that was in estrus was able to sit next to the alpha male as he modeled on all five days, with three high ranking males (NT, LH, LC, WO) and one juvenile female (WN) observing within a meter. Since only one piece of cereal was presented in the apparatus per trial, there was no opportunity for scrounging food from the alpha males.

**Table 1:** Method acquisition chart.

Social Group	Subject	Model Group	Sex	Age	Rank	Acquisition Order	Total No. of Trials	Total Lift	Total Slide	Percent Fidelity	Method Score
Lift	Ozzie *(OZ)*	Lift	M	20	H	model	859	858	1	99.88%	0.00
Lift	Nate *(NT)*	Lift	M	4	H	1*	672	663	9	98.66%	0.01
Lift	Luther *(LH)*	Lift	M	3	H	2*	489	486	3	99.39%	0.01
Lift	Lucas *(LC)*	Lift	M	8	H	3*	783	762	21	97.32%	0.03
Lift	Nancy *(NN)*	Lift	F	23	M1	4	69	63	6	91.30%	0.09
Lift	Wookie *(WO)*	Lift	M	4	M1	5*	121	120	1	99.17%	0.01
Lift	Wilma *(WL)*	Lift	F	10	M1	6	87	87	0	100.00%	0.00
Lift	Winnie *(WN)*	Lift	F	24	M2	7	31	27	4	87.10%	0.13
Lift	Lancey *(LA)*	Lift	F	6	L	8	3	3	0	100.00%	0.00
Lift	Lark *(LR)*	Lift	F	6	L	9	1	1	0	100.00%	0.00
Lift	Lulu *(LL)*	Lift	F	24	L	10	16	16	0	100.00%	0.00
Lift	Ike *(IK)*	Lift	M	34	M1	11	102	101	1	99.02%	0.01
Lift	Winter *(WT)*	Lift	F	4	M2	12	52	51	1	98.08%	0.02
Lift	Nicole *(NI)*	Lift	F	8	L	13	148	123	25	83.11%	0.17
Lift	Nadia *(ND)*	Lift	F	7	L	-	-	-	-	-	-
Slide	Mason *(MS)*	Slide	M	10	H	model	562	1	561	99.82%	1.00
Slide	Star *(ST)*	Slide	F	34	H	1*	822	9	813	98.91%	0.99
Slide	Scarlett *(SL)*	Slide	F	3	H	2*	124	1	123	99.19%	0.99
Slide	Snarf *(SN)*	Slide	M	4	M1	3*	623	3	620	99.52%	1.00
Slide	Sammie *(SM)*	Slide	F	12	M1	4	152	6	146	96.05%	0.96
Slide	Bias *(BI)*	Slide	F	21	M2	5	96	4	92	95.83%	0.96
Slide	Benny *(BE)*	Slide	M	4	M2	6	111	21	90	81.08%	0.81
Slide	Gonzo *(GN)*	Slide	M	2	M1	7	82	19	63	76.83%	0.77
Slide	Beeker *(BK)*	Slide	F	2	M1	8	55	2	53	96.36%	0.96
Slide	Mango *(MG)*	Slide	F	41	L	-	-	-	-	-	-
Slide	Bailey *(BY)*	Slide	F	8	L	-	-	-	-	-	-
Slide	Gretel *(GR)*	Slide	F	4	L	-	-	-	-	-	-

Social information is provided for each subject and presented by group in acquisition order. Asterisks (*) indicate subjects that were separated from the group on day 7.

Following the Observation Phase, a 1-hour, Open Diffusion Test session was conducted on each of seven days, with the whole group present and allowed access to the Doorian. In this phase, 13 out of the 14 observer subjects in the Lift group collected food from the apparatus. All 13 of these subjects used the lift rather than the slide method (binomial test: *p* = 0.0002; see Table 1) for the majority (i.e.>50%) of their trials (range 83.11 – 100% lift). The one subject who never accessed the apparatus was one of the lowest ranked females in the group.

In the Slide group, 8 out of the 11 observer subjects collected food from the apparatus and all used the model's slide method (binomial test, *p = *0.008; see Table 1) for the majority of their trials (range 76.83 – 99.52% slide); Three of the lowest ranking females in this group never attempted to collect food from the apparatus.

**Figure 3:**
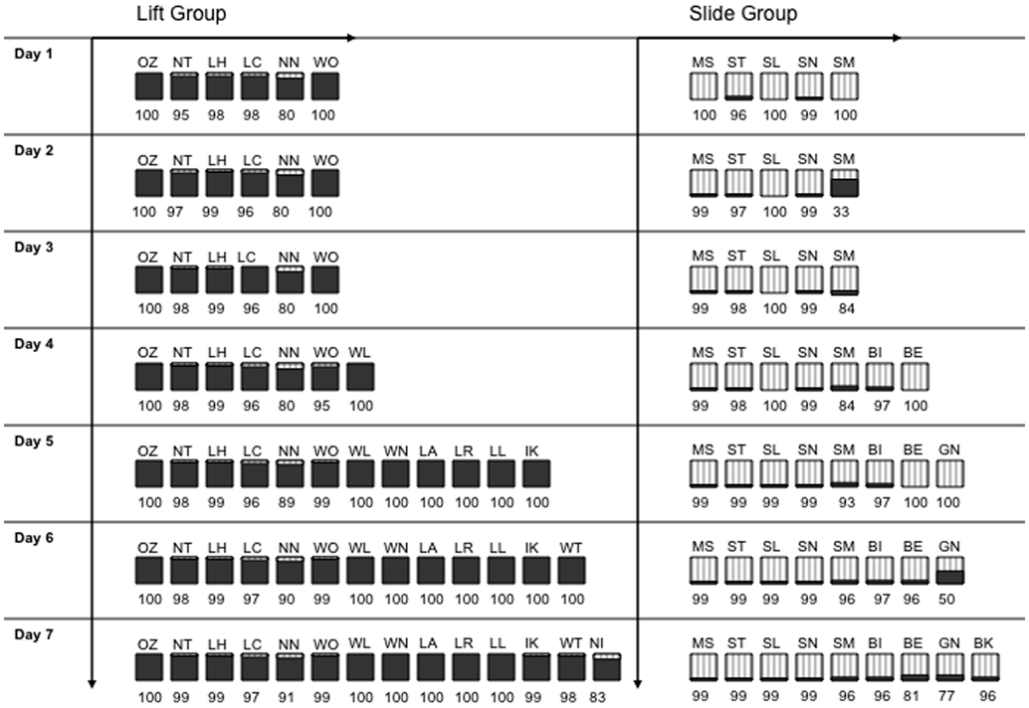
Group diffusion chart. Each square represents a subject, with the subject's code above and cumulative percent fidelity below. Subjects enter the chart on successful gaining of food fro the Doorian. Gray indicates the lift method and striped indicates slide. Left to right arrows indicate the order of acquisition beginning with the models (OZ and MS), and top to bottom arrows indicate the progression of days. The first letter of each code indicates to which matriline an individual belongs, and therefore also indicates relatedness. Note that NT, LH, LC, and WO were absent on day 7, thus there scores represent at total of 6 day.

Since both techniques were used in both groups, a ‘slide preference score’ was calculated as: the number of slide actions divided by the total number of slide + lift actions. (Table 1). The Slide group showed a significantly higher median slide preference score (0.96) than the Lift group (0.01) (two-tailed Mann-Whitney U Test: U = 0, z = −3.80, *p*<0.0001, n_A_ = 13, n_B_ = 8, see Figure 3). All subjects that collected food from the apparatus used their group specific method on the very first trial. Each of the 21 subjects subsequently maintained 76.8% fidelity or more for the group method, with 17 out of the 21 maintaining 90% or higher fidelity for their method.

However, while 4 subjects exhibited 100% fidelity to their method, seventeen of the successful 21 monkeys discovered the alternative action to that seeded in the group, performing it a median of 4 times. Nearly half of these seventeen subjects discovered the alternative method within the first 20 trials. Despite this ‘corruption’, none of these subjects performed more than 25 trials in total of the alternative method out of hundreds of later trials (Table 1). After their first discovery of the alternative method, the median fidelity remained as high as 99.03% (range 77.78%–100%; Table 1).

Weekly 30-minute ‘food scans’ were collected, in which subjects' order of access to a food tray was recorded. These data were used to assess the relative rank of group members (high, medium, or low ranking). This method has been used at the Living Links Capuchin Lab for over ten years and is generally a good indicator of group rankings [40]. Additionally, a ‘perceived-rank questionnaire’ was also given to three researchers within the capuchin laboratory to confirm the ranks derived from the food scan data. The order of acquisition of the techniques in each group was well predicted by relative rank order (Spearman's rho = 0.81, *df* = 19, *p*<0.001; Table 1). In both groups, only the highest ranked individuals gained access to the apparatus in the first three days, so those individuals (indicated by * in Table 1, and excepting the models) were locked in the front inside home area on day seven to prevent them from manipulating the apparatus and allow lower-ranking individuals access, mimicking a natural situation in which low rankers find such a foraging source on later occasions when higher-ranking performers are absent.

## Discussion

The majority of individuals in both groups showed a strong and sustained preference for the method demonstrated by the models. These results complement the growing number of reports that monkeys copy from others with greater fidelity than previously thought [29], [30], [31], [34], [35], and expands upon the few fully documented studies to seed different learning opportunities in an open group context with monkeys [33]. Chimpanzees are the only other primates for which open group diffusion experiments have yet been completed, offering parallel evidence for faithful transmission of alternative foraging techniques [41], [42], [43].

The only monkey diffusion study prior to our own, by Price and Caldwell [33], utilized a video-taped model to show a group of three and a group of four colobus monkeys either a simple push or pull technique for collecting food from a foraging apparatus. Their study showed that multiple individuals could learn together, but the limited number of subjects did not provide information about how the foraging behavior spread through colobus society. Use of video-taped models meant that social tolerance was not a variable affecting transmission, as it is in any study with live models. In contrast, the current study presented a more ecologically valid simulation of the nature of wild populations of capuchin monkeys [22], [23], as it involved two large social groups with subjects varying considerably in age, sex, relatedness and rank. Additionally, the limited visibility and proximity to others in the wild was mimicked by utilizing an environment where subjects had a choice to remain inside, away from the demonstrations in the outdoor area, or to come closer to observe. This likely assisted lower ranking individuals in their attempts to approach the foraging device when dominant individuals chose to leave, since their attempts could not be monitored by the dominants under these circumstances.

All subjects observed the alpha male models at least once throughout the observation phase. Since we cannot determine whether the subjects learned from these demonstrations alone, or from multiple individuals, we do not make any claims for observer preferences. Instead, we focus here on the chain of acquisition, since social ties and tolerance likely played a strong role in this process. In the initial observation phase of our study, only the highest ranking individuals were able to approach and watch the demonstrator within 1 meter. Thereafter, these same individuals were the first to manipulate the Doorian to obtain food, and largely did so with the same technique as the model. Capuchin monkeys exhibit a more relaxed social structure than other monkey species, such as Japanese macaques [10], [38], yet the order of acquisition of techniques suggests that rank played a strong role in the transmission process and in opportunities for learning.

A particularly intriguing finding was that during the first four days of the open diffusion phase, nine of the twelve high-ranking subjects discovered the alternative technique, and subsequently lower-ranking individuals observed the alternative technique in addition to the originally modeled one. Despite these interspersed ‘corruptions’ to the group's behavioral norm, all 21 subjects performed the group specific method on the first trial, and 17 of the 21 (81%) later discovered the alternative method yet continued to faithfully prefer the principal group technique. These results are consistent with more recent findings in white-faced capuchins in the wild, which indicate that these monkeys will conform to the foraging preferences of their closest social partners, despite having the knowledge of alternative techniques [23]. To our knowledge this is the first experimental evidence for such conformity in monkeys.

While there exist countless differences between human and animal cultures, the drive to act like others may be one of the most universal similarities between them [38]. In attempts to distinguish human from animal culture, some have emphasized imitation as a necessary prerequisite for cultural complexity, as it is the most faithful form of copying [44]. However the need for imitation may be overestimated, and learning about object movements (e.g. ‘door lifts’ versus ‘door slides’) may be sufficient for copying the technique of another individual. We cannot distinguish between such imitative and emulative processes in our study. To date, there is limited evidence for behavioral copying of any kind in capuchins and other monkey species [21], [22], [30], [31], [33], [35], [45], [46], which is why it is particularly surprising to find the level of fidelity to the group technique observed in this study. This level of conformity may be reinforced by the rewards obtained once an individual opts for one option over others [47] but in our study it was clearly shown to be initiated through social learning. ‘Conformity’, in the sense of social learning over-riding individual learning, has been shown in rats [48], but that study did not embed models in a whole group where a ‘copy the group norm’ bias is possible, as in our study. Conformity in such a group context despite knowledge of a viable alternative (in our study, the technique common in the other group) remains documented to date only in chimpanzees [49], humans [50] and in capuchins in the present study. Such potent social effects could offer strong underlying support to launch and sustain group specific traditions in the wild.

## Materials and Methods

### Subjects and Housing

#### Ethics statement

This study was conducted at the Living Links Capuchin Laboratory at the Yerkes National Primate Research Center in Atlanta, GA, USA, from May 25 to June 13, 2008. The YNPRC is fully accredited by the American Association for Accreditation of Laboratory Animal Care. This study was reviewed by the YNPRC veterinary staff to ensure that the social and physical conditions did not cause any suffering or stress for the monkeys, and was approved by Emory University's Institutional Animal Care and Use Committee (IACUC).

All individuals in two groups of capuchin monkeys (*Cebus apella*) served as subjects (N = 27) and all were naïve to the test apparatus. The Lift group consisted of 15 individuals: 3 adult males, 8 adult females and 4 juveniles, ranging in age from 3 to 34 years (median = 8 years). The Slide group consisted of 12 individuals: 1 adult male, 5 adult females and 6 juveniles, ranging in age from 2 to 41 years (median = 6 years). The alpha male from each colony served as the model for his respective group, with the remaining members serving as observer subjects. Both groups were housed in the same building, where they were visually but not acoustically separated, with combined indoor and outdoor enclosures measuring 25 and 31 m^2^, respectively (Figure 2).

Participation in the study meant that a subject either had to observe another monkey collect food from the apparatus or approach the apparatus themselves to collect food from it. Since subjects had access to all areas of their home enclosures during testing, participation was voluntary. On Day 7 of the Open Diffusion condition, specific high-ranking individuals were prevented from monopolizing the apparatus and were not allowed outside for the entire testing hour. They were given regular access to the front indoor area of their enclosure, while everyone else had access to the back of the indoor area and full access to the outdoor enclosure. Subjects were never food or water deprived. Tests commenced approximately 1 hour after the afternoon feeding inside.

### Apparatus

The same foraging apparatus used by Dindo and colleagues [30] was employed in this study. The apparatus was constructed from Lexan and measured 28×28×28 cm. The front of the apparatus faced the mesh enclosure, and was accessible to the monkeys, while the back of the apparatus faced the experimenter, who sat behind it (Figure 2). The back of the apparatus was open to allow the experimenter to place food on a hidden tray. The tray could be accessed by a subject from the front of the apparatus by either (1) lifting or (2) sliding an opaque door (Figure 1). Each trial consisted of a subject opening the door by either method and food being collected from the tray. In the lift condition, the door returned to the start position by gravity once it was released. In the slide condition, the experimenter returned the door to the closed position by pushing a pin at the back of the door. Pieces of cereal were used as food rewards.

### Model Training Procedure

The alpha males of each group (OZ & MS) were selected as models since their high rank would ensure that they would not be displaced by other group members during the Observation Phase. For three consecutive days, OZ and MS were briefly separated from their groups for training. The experimenter (MD) demonstrated the lift or slide method to the respective model and allowed the model to take the cereal from the food tray inside the apparatus. Both models began collecting food using the method presented to them and were considered proficient models after these training sessions, and so the Observation Phase began on the following day.

### Observation Phase

The Observation Phase consisted of presenting the apparatus along the mesh of the outside enclosure to the trained models, OZ and MS. Each observation session consisted of 50 trials, where, due to the models' alpha-status and ability to monopolize a resource, only the model had the opportunity to manipulate the door. When the model was present, no other monkeys were able to handle the apparatus. Each model demonstrated only his respective trained method. If the model walked away at any point, the apparatus was pulled back, out of the reach of other group members, until he returned. It took between 9–10 minutes in total to complete all 50 trials for each observation session, and only one session was given per day per group. Each group received a total of five observation sessions before the Open Diffusion Test Phase began.

### Open Diffusion Test Phase

One hour before each Open Diffusion Test session, the afternoon food trays were presented inside. Food trays included oranges, bread, and vitamin formula and were given daily in the afternoon. Tests took place after 6 p.m. to avoid the summer heat, as well as to give at least an hour's break between feeding and testing. Just as in the Observation Phase, the apparatus was presented to each group in the same place every day (Figure 2), and the model demonstrated how to collect food from it; however, unlike the Observation Phase, if the model left the apparatus, other individuals were allowed to manipulate it and collect food.

One Open Diffusion Test session was conducted per day per group for approximately 1 hour per session. Tests were at least one hour long, so that no one individual was likely to monopolize the apparatus for the entire test session. In total, seven days of testing per group were conducted to provide a generous number of trials, in order to examine if the monkeys would remain faithful to the foraging methods they observed other group members using. Multiple days and hundreds of trials were necessary in order for the majority of the group to learn to forage from the apparatus, which made it possible to determine if a group tradition formed for a specific way of foraging.

### Data Collection and Analyses

All tests were recorded on video from two locations (Figure 2). The first camera was situated behind the experimenter and provided a view of the entire enclosure. The resulting video tapes were coded for the identity of individuals observing each trial, and their proximity to the apparatus within 1 meter. The second camera filmed the front of the apparatus to record the identity of the subject per trial and the method used per trial. The experimenter also dictated the identity of subjects, methods used, and those observing each trial. This information as well as the two tapes per test were used for coding. One test was selected at random and was coded for intra-observer reliability for the method used and identity of the subject. The kappa for agreement was 0.944, indicating a high level of agreement.
